# Plasma Modeling and Prebiotic Chemistry: A Review of the State-of-the-Art and Perspectives

**DOI:** 10.3390/molecules26123663

**Published:** 2021-06-16

**Authors:** Gaia Micca Longo, Luca Vialetto, Paola Diomede, Savino Longo, Vincenzo Laporta

**Affiliations:** 1 Dipartimento di Chimica, Università degli Studi di Bari Aldo Moro, Via Edoardo Orabona 4, 70126 Bari, Italy; gaia.miccalongo@uniba.it; 2 Center for Computational Energy Research, DIFFER—Dutch Institute for Fundamental Energy Research, De Zaale 20, 5612 AJ Eindhoven, The Netherlands; l.vialetto@differ.nl; 3 Faculty of Science and Engineering, Maastricht University, Paul Henri Spaaklaan 1, 6229 GS Maastricht, The Netherlands; p.diomede@maastrichtuniversity.nl; 4 Istituto per la Scienza e Tecnologia dei Plasmi, CNR, Via Amendola, 122/D, 70126 Bari, Italy; vincenzo.laporta@istp.cnr.it

**Keywords:** Miller-Urey experiment, prebiotic chemistry, plasma kinetics, electrons prebiotic molecules

## Abstract

We review the recent progress in the modeling of plasmas or ionized gases, with compositions compatible with that of primordial atmospheres. The plasma kinetics involves elementary processes by which free electrons ultimately activate weakly reactive molecules, such as carbon dioxide or methane, thereby potentially starting prebiotic reaction chains. These processes include electron–molecule reactions and energy exchanges between molecules. They are basic processes, for example, in the famous Miller-Urey experiment, and become relevant in any prebiotic scenario where the primordial atmosphere is significantly ionized by electrical activity, photoionization or meteor phenomena. The kinetics of plasma displays remarkable complexity due to the non-equilibrium features of the energy distributions involved. In particular, we argue that two concepts developed by the plasma modeling community, the electron velocity distribution function and the vibrational distribution function, may unlock much new information and provide insight into prebiotic processes initiated by electron–molecule collisions.

## 1. Introduction

Earth’s chemical history and the origin of life are two of the most crucial topics in astrochemistry and astrobiology [1,2,3,4]. Two main theories [5,6] have been proposed regarding the emergence of life on Earth: (i) exogeneous delivery and (ii) endogenous synthesis. According to the first theory, prebiotic molecules have reached the Earth’s surface by means of comets, meteors and asteroids. The so-called delivery, that is, the actual transport of molecules from space to Earth, represents the key point of the Panspermia theory [7,8,9,10,11]. It was demonstrated that carbonate and sulfate micrometeoroids (especially calcite and anhydrite) may act as good prebiotic molecule carriers [12,13,14], in view of the well-known association between carbonates/sulfates and organic matter presence [15,16,17,18]. Recently, the role of the chemical composition of the primordial atmosphere was pointed out, in the context of the atmospheric entry of the aforementioned carbonate and sulfate micrometeoroids [19].

On the other hand, the endogenous synthesis approach, developed in the 1950s and 1960s, speculates that primordial atmospheres of terrestrial planets were filled up with methane, ammonia and hydrogen, and it theorizes that the primordial clouds provide the necessary chemical and energetic conditions to activate small molecules and link them into bigger molecules, in order to synthesize the building blocks of life. The topic of early Earth’s atmosphere as a source of prebiotic matter comes from the Miller-Urey experiments [20,21,22], partially modeled on Oparin’s hypothesis of reducing primordial atmosphere [23]. So, the endogenous theory states that the synthesis of organic molecules occurred directly on Earth starting from simple parent molecules. Actually, a third theory concerning the origins of life has been proposed, related to the production of organic molecules and even amino acids in hydrothermal vents [24].

**Figure 1:**
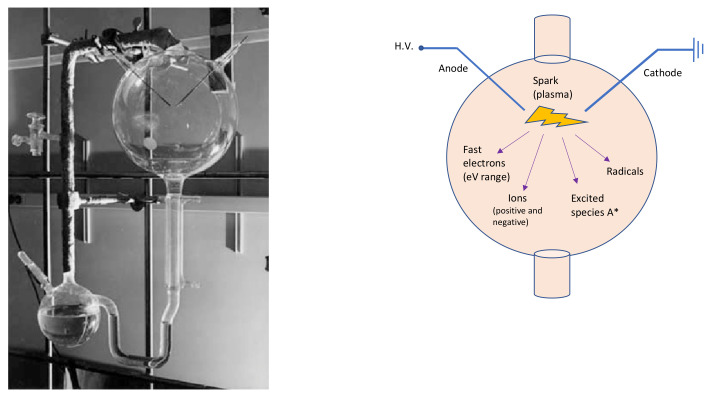
On the left, the Miller-Urey experimental apparatus. The lower flask contains the primordial ocean model, while, through the gas inlet/outlet on the right, the rest of the apparatus is filled with the strongly reducing primordial atmosphere, based on the knowledges of Miller’s times. The third neck of the lower flask is used to sample the liquid for analysis. The higher balloon displays the spark gap. Photo adapted from [25]. On the right, a schematic sketch of the upper glass flask. A* symbolizes the excited species.

Miller-Urey’s experimental apparatus (left side of Figure 1) consisted of two glass flasks: the lower one was partially filled with liquid water—to mimic the primordial ocean—, and a gas mixture composed of methane, ammonia and hydrogen—reproducing the primordial atmosphere—filled the other balloon. In this primordial gas, Miller placed two electrodes to simulate a lightning-like electric discharge. Therefore, Miller and Urey recreated the disequilibrium chemistry conditions by applying an electrical discharge to the gases, which probably formed a highly reducing primordial atmosphere. The two flasks in the apparatus were connected in a hydrological cycle, which allowed it to mimic rain after the electrical discharge. The analyses demonstrated that, after the experiment, the primordial ocean was rich in carbon compounds, which contained significant amounts of amino acids. Miller and Urey demonstrated that organic molecules can form spontaneously from inorganic components, in such primordial reducing environments. It can be stated that the Miller-Urey experiment is the first abiotic synthesis of organic compounds, under simulated primordial Earth conditions, in the context of the studies on the origin of life.

Although recent progress in astrochemistry and astrobiology suggests a role of increasing importance of the activation process by UV radiation and shock waves [26,27], the following considerations must be taken into account: (*i*) The Miller-Urey experiment represents a milestone in prebiotic chemistry, from the conceptual, historical and pedagogical point of view; therefore, a better understanding of the experiment can add new interesting results in prebiotic studies; (ii) The chemical activation by shock waves, like that produced by meteors, proceeds, in the case of high Mach numbers, through the ionization of the gas. Fast energy electrons are still responsible for the chemical activation of the neutral gas. A better understanding of the Miller-Urey experience phenomenology can also help, therefore, in the scenarios of chemical activation by shock waves; (iii) The activation mechanism by electric discharge needs to be better understood in light of the growing knowledge of electrical phenomena in planetary atmospheres, such as those of Venus and Titan.

With respect to the studies carried out in the few years following Miller experiment, considerable progress has been made in terms of theoretical and methodological understanding (see [28,29] and references therein). Moreover, hypotheses regarding the composition of the primordial terrestrial atmosphere have changed considerably.

The composition and depth of the primordial atmosphere are the subject of a lively debate in the planetology community. Dating back to Oparin [23] and Haldane [30], most scientists believe that, at the very least, the primordial atmosphere was enriched in reducing low-molar-mass components (methane, ammonia and even hydrogen). Additionally, there is no reason to assume that an early Earth atmosphere (a mixture of components with very different molar mass, like nitrogen and hydrogen) should have uniform composition in its lower and upper regions: probably, the early atmosphere was enriched in low mass components at high altitudes.

Hypotheses regarding the chemical composition of the Earth’s earliest atmosphere are numerous. Moreover, it is believed that there were many atmospheres over the vastness of Earth’s geological time [31]. Geologically based argumentations, that treat the atmosphere as outgassed from solid Earth, indicate that the Earth’s primitive atmosphere was composed mostly of H${}_{2}$O, CO${}_{2}$ and N${}_{2}$, with traces of CO and H${}_{2}$ [32,33,34].

Since the 1980s, geoscientists have been disputing Miller’s hypothesis of highly reducing primordial atmosphere [35]. Many experiments were carried out using CO and CO${}_{2}$ atmospheres [36,37], showing that a CO-dominant atmosphere might have produced organic compounds; however, the synthesis of organic compounds by the action of electric discharges in neutral gas mixtures is much less efficient, in this case. Results presented by Cleaves et al. [38] demonstrated that neutral atmospheres can produce amino acids in much higher amount than previously thought. A complete and exhaustive review about the origin and conditions of the Earth’s earliest atmospheres can be found in the work by Zahnle et al. [39], with particular attention to the issue concerning the origin of life (∼4.6–4 Gys ago).

Under the hypotheses that the primordial Earth’s atmosphere consisted predominantly of CO and CO${}_{2}$, the chemical channels previously considered for a reducing atmosphere (based on the preliminary production of the highly reactive HCN molecule) must be reformulated and subjected to new studies, bearing in mind that other components, even minority ones, can play a role in subsequent chemical reactions. Although theoretical studies on the CO and CO${}_{2}$ system in the context of the Miller-Urey experiment are advanced [40,41,42], one aspect of these studies is still rather primitive, namely the role of electrons in the plasma (a word indicating an ionized gas introduced by I. Langmuir [43]) in producing the first excited and radical species and ions, which later enter several chemical channels to form prebiotic molecules. Studies of chemical kinetics performed in the case of gas discharges with chemical composition enormously simpler than the one used in Miller-type experiences have shown that ionization and excitation processes have an extremely complex and interesting kinetics. These studies show that the efficiency of radical formation can be much greater than the one observed in the much more phenomenological studies devoted to Miller’s experience so far.

Several studies actually considered the chemical activation *via* plasma electron of planetary atmospheric compositions with a prebiotic perspective. For example, plasma chemistry has been studied in electric discharges fed with chemical compositions compatible with the present atmosphere of Titan [44,45,46,47,48] and for Venus and Jupiter atmospheres [49].

**Figure 2:**
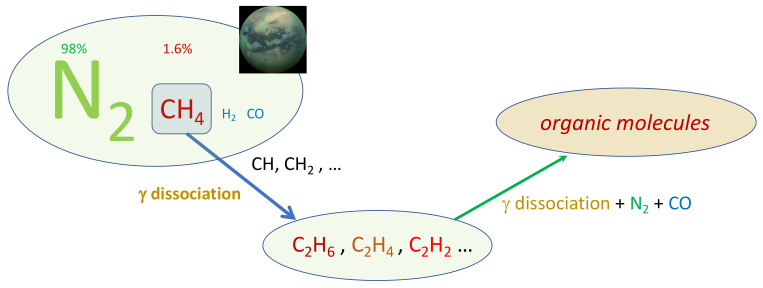
Prebiotic chemistry in Titan atmosphere.

Titan resembles the terrestrial planets, and particularly Earth, as it is the only other body in our solar neighbourhood with liquid reservoirs on its surface. The familiar role of water in our atmosphere is replaced by methane, which survives as a liquid at the extremely low temperature conditions at the surface (−180 ${}^{{}^{\circ}}$C). The scheme in Figure 2 reports the chain of reactions of prebiotic chemistry in Titan atmosphere.

The fact that plasma chemistry might be relevant to the interpretation of the Miller-Urey experience was mentioned in the 1978 Libby review [50]: in particular, this review discusses the capacity of the methyl cation CH${}_{3}^{+}$, formed by the ionization of methane, to produce higher organic species by ion-neutral reactions. At that time, however, there was no possibility to model the kinetics of complex molecular plasma like ionized methane. This was developed more recently, mostly for the study of material science processes where CH${}_{4}$ is ionized in order to ultimately fix carbon into diamonds [51,52], or carbon nanotubes [53].

All these considerations lead to the the idea of exploring prebiotic molecular mechanisms in the early stages of the production of new chemical species from a plasma kinetics point of view, highlighting therefore the importance of electrons and excited molecules in chemical channels.

The aim of this paper, in the framework of the endogenous synthesis, is to show how recent developments in the theoretical modeling of chemical reactions and transport phenomena, in weakly ionized gases and in thermodynamic non-equilibrium conditions, can shed a new light on the studies on the synthesis of prebiotic molecules in the primordial atmosphere. Actually, in our view, contributions from delivery mechanisms are not completely ruled out, since they can provide useful monomers which can react with active species produced from the native medium. Our primary goal is to fill the gap between plasma kinetics and prebiotic chemistry. We do believe that the original idea of bringing together two “distant” theories may represent a solid base for future important developments.

The paper is organized as follows: in Section 2, we introduce non-equilibrium plasma kinetics and, in particular, the role electrons can have in understanding primordial chemical processes. In Section 3, we present the kinetics of molecular activation by plasma electrons in plasma discharges, with the techniques, which have good chances to be suitable even for the complex molecules in prebiotic chemistry. Using a consistent set of accurate cross sections for many elementary processes, we show a realistic distribution of the kinetic energy of free electrons, assuming a realistic chemical composition for the gas for two primordial atmosphere compositions frequently discussed in previous studies. Section 4 reports an overview on ab initio calculations of cross sections. Final considerations and outlooks are presented in Section 5.

## 2. Tools from Plasma Kinetics

Plasma kinetics is the study of chemical reaction rates in ionized gases [54]. It is a very active field of study with many applications in microelectronics, aerospace, medicine and astrochemistry, just to name a few. Thanks to many previous studies [55,56,57], we have knowledge of the physical conditions of a plasma associated with natural phenomena, such as for example lightning discharges.

Plasma kinetics was born with the intent to understand more quantitatively the physics of systems with a considerable degree of non-equilibrium, in particular ionosphere chemistry. Over time, it has developed its own calculation and prediction methods, principally coming from statistical mechanics, which are very specialized with respect to those used in chemical kinetics of gases and in chemical kinetics in general.

In such non-equilibrium (sometimes called “low-temperature”, or “cold”) plasmas, electrons have a much greater kinetic energy (from few eV to few tens of eV) than that of atomic and molecular neutral species, and distributions of speed and energy are not trivial. These peculiarities are produced by energy flows and energy exchanges within the system, whose main origin is the acceleration of electrons (and, to a lesser extent, of ions) by the electric field; the electric field is applied to the plasma by means of two electrodes at different electrical potential. Electrons maintain a high average energy because of the slow processes of dissipation of electronic kinetic energy, due to the unfavorable mass-ratio between electrons and other species (which slows down energy transfer in elastic collisions), and the small cross sections for inelastic processes (compared to the corresponding elastic ones). The energy distribution of free electrons is therefore a complex function that cannot be reduced to the Maxwell-Boltzmann distribution, as normally assumed in the chemical kinetics of gases. This is of paramount importance, because the violation of the Maxwell-Boltzmann distribution rules out the renowned Arrhenius law, used in kinetic studies of prebiotic processes.

In view of the knowledge produced in plasma science, there is little doubt that the earlier studies on the kinetics of the Miller-Urey experience must be reconsidered. The primary chemical activation of the gas is associated with chemical dissociation and ionization of small monomers molecules present in the original Miller-Urey mixture and modern variants: these molecules are CO${}_{2}$, CO, H${}_{2}$, NH${}_{3}$, CH${}_{4}$, H${}_{2}$O, NO, and a few others. While a huge number of studies has been produced concerning chemical reactions of activated monomers to produce amino acids, aldehydes and sugars, very little has been studied in the direction of a better understanding of the primary chemical activation. In this process, the presence of high energy free electrons (whose share is to be determined), fast ions, quantum mechanics and statistical mechanics of activation are new fields where progress occurs on a month-by-month basis.

Very effective deterministic, stochastic and hybrid methods have been developed over the years to determine the electron energy distribution, typically as a function of the reduced electric field Ɛ/N (Ɛ is the magnitude of the electric field, *N* is the gas number density) and the neutral gas composition [58,59,60,61,62,63,64,65,66]. The strong gap between the average energy of the electronic component and that of the neutral component leads in turn to a chemical kinetics that requires adequate models, so far not very much used outside plasma electronics, aerospace and a few studies on the chemistry of the interstellar medium. These models root in the determination of reaction channels following the flow of energy provided by free electrons, which constantly produces reactive species such as ions, free radicals and excited states of atoms and molecules. Such a state-of-the-art entails concrete possibilities that the know-how implemented and validated in the field of plasma kinetics can lead to remarkable results if applied to the study of those systems in astrobiology where similar circumstances are met. Taking into account specifically the chemical peculiarities of Miller-Urey’s experience, particularly in the new formulations that are emerging, these aspects will be illustrated in the following sections by means of two case studies, which, however, only aim to represent a small example of the great variety of useful research that can be performed on the same basis.

## 3. Kinetics of Molecular Activation by Plasma Electrons

The theoretical study of reaction rates involving molecules has progressed remarkably since the first formulation of Miller-Urey’s experience. A particularly important technique, developed in parallel in plasma kinetics and in astrophysics, is the so-called state-to-state (STS) approach. In this formulation of chemical kinetics, the individual excited levels of molecules are treated as individual species, with their own characteristic reaction data. The STS is able to comply with the fact that the specific reaction rates of the different excited states of most molecules may differ by orders of magnitude. Furthermore, the distribution of populations of the different levels in a system far from equilibrium, such as a cold plasma, can differ considerably from the results obtained in statistical mechanics applied to equilibrium systems, that is, the Boltzmann distribution.

As a result, the mole fraction of tens or hundreds, sometimes thousands, of individual “components”, $\chi_{i}$, must be calculated by solving extended systems of ordinary differential equations (ODE’s) of the form

(1) $$\frac{d}{dt}\chi_{i}=R_{i}\left(\chi_{j}\right),$$

addressed in this context by the term Master Equation (ME). $R_{i}$ is the reaction rate for the *i*-th STS component, which is a function potentially of all of the χ’s: it includes many terms describing elementary reactions between STS components. The ME is tricky to solve accurately, since the different contributions to its r.h.s. may differ by orders of magnitude, making the system of ODEs so-called “stiff” in computational terms. Nevertheless, many fruitful and concrete applications of this approach have been demonstrated for reactive fluids, lasers and electric discharges [67,68,69,70,71,72].

An example is provided by the chemical activation of CO${}_{2}$, which is a very stable molecule, often abundant in the atmospheres of rocky planets. It appears that some processes, under the conditions of a primordial atmosphere, can activate chemically this molecule with the help of physical energy in the form of, for example, kinetic energy of the free electrons. In this respect and in recent years, great progress has been made in the theoretical understanding of the chemical plasma activation of CO${}_{2}$, due to the importance that this process plays in the CO${}_{2}$ recycling study for the mitigation of the climatic consequences of CO${}_{2}$ accumulation in the atmosphere [73,74].

Since the most effective dissociation path of CO${}_{2}$ is through the asymmetric stretching vibrational mode, it is necessary to distinguish the 22 vibrational levels of this mode [73]. Electron-impact vibrational excitation processes, the so-called “eV-processes”, however, can only pump energy into the lowest vibrational levels, say the first 4 or 5, because cross sections of the direct excitation of higher levels are very low:

(2) $$e+{\mathrm{CO}}_{2}\left(v\right)\to e+{\mathrm{CO}}_{2}\left(v+1\right)\;.$$

Subsequently, CO${}_{2}$ molecules reach the dissociation limit with the help of a different process, named VV${}_{1}$, where a vibrationally excited CO${}_{2}$ molecule interacts with a more excited one pushing it to higher excitation:

(3) $${\mathrm{CO}}_{2}\left(v=1\right)+{\mathrm{CO}}_{2}\left(w\right)\to {\mathrm{CO}}_{2}\left(v=0\right)+{\mathrm{CO}}_{2}\left(w+1\right)\;.$$

The mechanism in Equation (3), proposed by Russian scientists about 40 years ago [75], pushes molecules up the internal energy ladder until they reach the dissociation threshold, *i.e.*:

(4) $${\mathrm{CO}}_{2}\left(v=1\right)+{\mathrm{CO}}_{2}\left(w=21\right)\to {\mathrm{CO}}_{2}\left(v=0\right)+\mathrm{CO}+O\;.$$

The process described by the reactions in Equations (2)–(4) is extremely effective in increasing the efficiency of activation, since the direct dissociation channel with electronic states as intermediates has an energetic cost in the order of 10 eV, whereas the energy cost for vibrational excitation is less than 1 eV.

**Figure 3:**
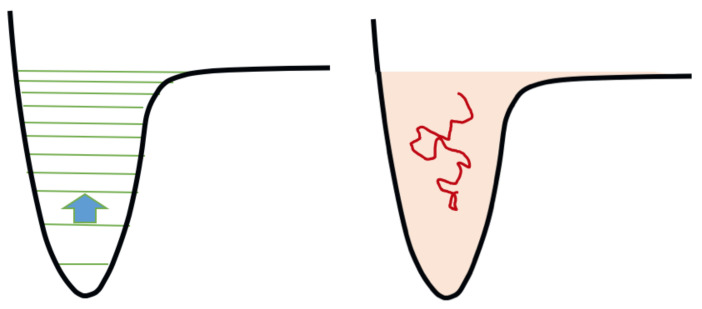
Symbolic representation of the state-to-state chemical kinetics and its equivalent in the continuum. On the left, the arrow represents a one-quantum collisional transition between two consecutive levels. On the right: the level manifold is approximated by a continuum of energy, the kinetics is sampled by Brownian motions, the energy transport is drift-diffusive. Drawing adapted from [76].

In the last few years, many researchers, including two authors of the present paper, aimed to achieve a better understanding of this mechanism using computer simulations. For example, a recent scheme is based on the characterization of the upwards flux in energy space, *J* [76,77,78]. *J* is a functional of the function f(ϵ), which is the distribution of molecules according to their internal energy ϵ. The theoretical basis of this approach is the relative smallness of energy difference between consecutive vibrational quantum states compared with the binding energy for the majority of molecules; that is, in many molecules the vibrational levels for a given electronic curve are numerous. For example, in the case of the nitrogen molecule, they are around 50 for the electronic ground state, depending on the potential energy curve assumed; a lower number, 15, holds for the hydrogen molecule. Most of the chemical processes between excited vibrational levels involve the exchange of no more than one quantum. We are therefore in the transition/continuum regime as schematically represented in Figure 3. Consequently, it is possible to formulate the problem by means of a diffusion equation, or more generally of a drift and diffusion equation, that is the Fokker-Planck equation:

(5) $$\frac{\partial f}{\partial t}=-\frac{\partial a_{1}\left(\epsilon \right)f}{\partial \epsilon }+\frac{1}{2}\frac{\partial^{2}a_{2}\left(\epsilon \right)f}{\partial \epsilon^{2}}\;.$$

It has been shown [76,77,78] that the two transport coefficients in Equation (5), namely the two jump moments $a_{1}\left(\epsilon \right)$ and $a_{2}\left(\epsilon \right)$, can be calculated starting from the rate coefficients of the chemical reactions involving the different vibrational states. The calculation is not simple and the use of formulas requires the evaluation of numerous data; nevertheless the calculation is possible and produces results that are reliable and comparable with those based on the explicit discretization of the internal energy. However, discretization requires very long computational times and can hardly be applied to future theoretical formulations of Miller’s experience, especially considering that in this experience complex species formed starting from monomers with numerous levels of internal energy are also involved.

**Figure 4:**
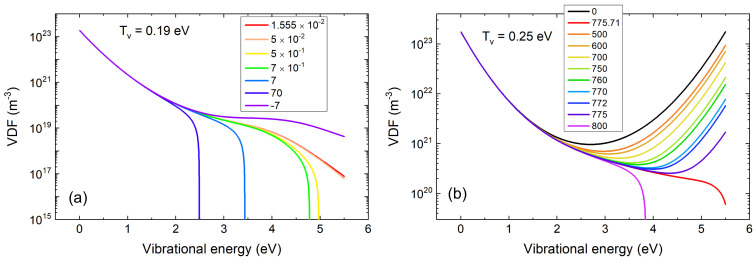
Calculated vibrational distribution function of CO${}_{2}$ molecules based on the functional method J[f] described in the text, in the case of a lower vibrational temperature $T_{v}=0.19$ eV (**a**) and an higher one $T_{v}=0.25$ eV (**b**). In the low $T_{v}$ case, the vibrational distribution has strong impact on the dissociation rate *J*. In the higher $T_{v}$ case, *J* becomes rather insensitive to the vibrational distribution. Figure adapted from [77].

The mathematics of diffusion, namely the continuity equation of the flux through the internal energy space, demonstrates that, at steady-state, the aforementioned flux functional *J*, whose expression is:

(6) $$J=b\left(\epsilon \right)f\left(\epsilon \right)-D\left(\epsilon \right)\frac{\partial f}{\partial \epsilon }\;,$$

where the internal energy drift b(ϵ) and diffusion D(ϵ) coefficients are:

(7) $$b\left(\epsilon \right)=a_{1}-\frac{1}{2}\frac{\partial a_{2}}{\partial \epsilon }\;,\;D\left(\epsilon \right)=\frac{1}{2}a_{2}\left(\epsilon \right)\;,$$

is a constant, that is, it does not depend on the internal energy ϵ. Furthermore, it can be shown that J[f] is the dissociation rate. f(ϵ) can be easily calculated by solving the Fokker-Planck equation based on rate coefficients of vibrational processes and the known value of *J* [78]. But, since *J* needs *f* to be calculated, the process is iterated until *J* is self-consistency attained, and the problem is solved. The behavior of *f* as a function of *J* can provide additional insight into the process of molecular dissociation. This is shown in the plots in Figure 4, which are based on calculations of the described type: in all cases, on the *x*-axis we set the internal energy, which goes from the value 0, corresponding to the zero-point energy of the quantum oscillator that describes the asymmetric stretching motion, to the dissociation energy. The plotted functions are precisely the internal energy distributions. The two plots differ in the value of $T_{v}$, the excitation temperature of the 0→1 transition.

Comparing the plot on the left in Figure 4 (lower temperature) with the one on the right (higher temperature) and observing the values of *J* corresponding to the different curves shown in each of the two figures, two completely different behaviors can be observed. While in the case of the lower temperature, the function can only be modified with considerable variations of the functional *J*, in the case of high temperature, completely different energy distributions are obtained for very small variations of the functional *J*. On the one hand, the low-temperature case exemplifies that the energy distribution determines the dissociation rate, especially in the high-energy region, which is closer to where the dissociation takes place. On the other hand, in the high temperature case, a regime is realized in which the functional *J*, and therefore the dissociation rate of the molecules, changes very little even in the hypothesis of large variations of the high energy part of the distribution.

This last result is very important in a reconsideration of the Miller-Urey experiment with an atmosphere rich in CO${}_{2}$. In such an atmosphere, locally produced plasmas may produce radicals from CO${}_{2}$ with a very high energetic efficiency, which was unforeseeable at Miller’s times.

A similar synergistic mechanism could speed up considerably the dissociation of other molecules relevant to modern studies of planetary atmospheres in a prebiotic perspective: one of those is methane (CH${}_{4}$). A continuum model for the internal energy flux in methane is completely lacking at present, but it is within reach of the present knowledge and methods and could advance the understanding of prebiotic chemistry, again, with the help of methods from plasma kinetics.

As regards electron kinetics, the characterization of the distribution of free electrons is fundamental to describe the thermodynamic state and its departure from equilibrium [65]. From the distribution function, it is also possible to calculate macroscopic quantities, such as electron swarm parameters [79], and electron impact rate coefficients. To calculate the electron distribution function, it is necessary to solve the Electron Boltzmann Equation (EBE). Several codes are available as open-source for calculation of the Electron Velocity Distribution Function (EVDF). Most of them are based on the two-term expansion of the EVDF in Legendre polynomials, such as BOLSIG+ [64] and LoKI-B [80]. At the expenses of computational time, other codes employ different numerical methods based on multi-term expansion of the EVDF in Legendre polynomials (e.g., the MultiBolt code [81]) and Monte Carlo (MC) simulations (e.g., the METHES code [82]), in order to obtain more accurate calculations.

**Figure 5:**
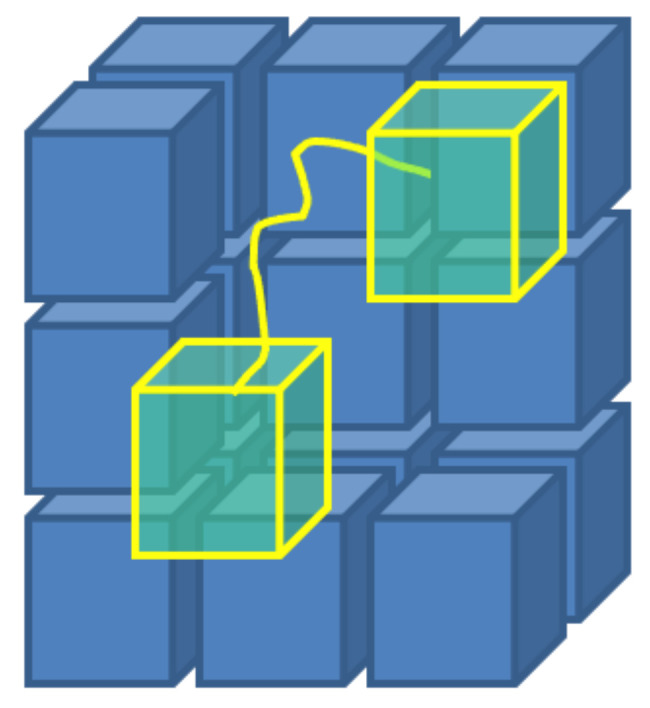
A scheme of the concept behind the Monte Carlo Flux method: stochastic simulations, that take place in the phase space, allow to calculate the transition frequencies $q_{ij}$ between sub-regions of the same phase space, obtained by arbitrary partition.

Recently, a code based on the Monte Carlo Flux (MCF) method [59] has been implemented and benchmarked by some of the present authors against two-term and multi-term Boltzmann solvers [66,83]. The MCF method provides detailed calculations of the EVDF through a variance reduction technique, that provides a highly computationally efficient solution of the electron transport problem, compared with conventional MC simulations. In MCF, the phase space of electrons is partitioned into cells, and the problem of electron transport is reformulated in the following form [65]:

(8) $$n_{i}\left(t+\Delta t\right)=\sum_{j}q_{ji}\left(\Delta t\right)n_{j}\left(t\right)-n_{i}\left(t\right)\sum_{j}q_{ij}\left(\Delta t\right),$$

where $n_{i}\left(t\right)$ is the number of electrons in the *i*-th cell at time *t*, Δt is the MC time step for calculation of the transition probabilities $q_{ij}\left(\Delta t\right)$. Hence, a set of MC simulations is performed to calculate the coefficients $q_{ij}$ by placing systematically sample electrons in the *i*-th cell and collecting the number of electrons in any *j*-cell after a time Δt [65] (Figure 5).

Then, the time evolution of the electron distribution is obtained by an iterative solution of Equation (8). As an alternative, the steady-state solution can also be calculated by means of an eigenvalue method [59]. The advantage of MCF is that it can calculate EVDFs with uniform statistical accuracy in low density regions that cannot be treated by the MC method. Moreover, in [83], the MCF method has also been extended to take into account the thermal velocity distribution function of gas molecules, together with an accurate description of rotational and vibrational excited states for a pure CO${}_{2}$ gas.

In the present work, MCF has been employed for simulations of electrons in mixtures of gases representative of the composition of the primordial Earth atmosphere [84]. In particular, two different compositions have been analyzed:

- A *highly reduced mixture of gases* including CH${}_{4}$, NH${}_{3}$ and H${}_{2}$O, with molar fractions of 0.7, 0.2 and 0.1, respectively. This composition is taken as representative of Miller-Urey-type experiments and it has also been considered in recent experimental investigation by Scherer and co-authors [85].
- A *mildly reduced mixture of gases* dominated by N${}_{2}$ and CO${}_{2}$, with traces of CO, H${}_{2}$O and H${}_{2}$. The molar fractions of the gases are 0.7 for N${}_{2}$, 0.2 for CO${}_{2}$, 0.05 for H${}_{2}$O, 0.025 for CO and for H${}_{2}$. This composition is representative of a prebiotic Earth atmosphere (around 3.8 Gy) at sea level, as suggested in the pioneering study by Kasting [35]. At higher altitudes, traces of O${}_{2}$ and O are also formed from photo-dissociation of CO${}_{2}$ [35]. However, for simplicity, this process has been neglected in the present study.

Here, the cross sections sets that are used as input for the simulations are illustrated for each gas. In this work, we include also superelastic collisions from vibrational states, i.e., collisions of electrons with excited states leading to de-excitation. The cross sections for these processes have been calculated with the formula of Klein-Rosseland [86].

- *Methane*: Cross sections for CH${}_{4}$ are taken from the Magboltz code version 11.9 developed by Biagi [87].
- *Ammonia*: Cross sections for NH${}_{3}$ are taken from the Hayashi database of the LXCat database [88].
- *Water vapour*: Cross sections for H${}_{2}$O are taken from Biagi’s code Magboltz version 11.9 [87].
- *Carbon dioxide*: Electron impact cross sections for CO${}_{2}$ are taken from the Biagi database of LXCat [89]. The elastic momentum transfer cross sections from the Biagi database is corrected to take into account the population of vibrational bending mode levels and quadrupole rotational collisions, according to Vialetto and co-authors [83].
- *Carbon monoxide*: Electron impact cross sections for CO are the ones from the Magboltz source code v 11.9 [87]. In [90], this set has been used for calculations of electron transport parameters in CO, with particular focus on the treatment of dipole rotational collisions. A Morse anharmonic oscillator is used for calculations of the energy of vibrational states, whereas a rigid rotator model is assumed for rotational levels [91].
- *Hydrogen*: Electron impact cross sections for H${}_{2}$ are taken from the IST-Lisbon database of LXCat [92].
- *Nitrogen*: Electron impact cross sections for N${}_{2}$ are taken from the IST-Lisbon database of LXCat [92].

**Figure 6:**
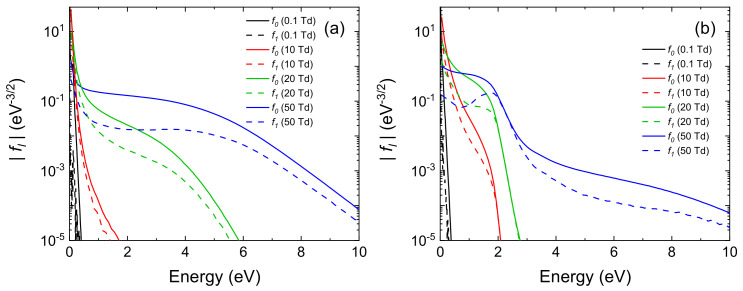
Zeroth (solid line) and first order (dashed line) Legendre polynomials coefficients calculated from MCF at different reduced electric fields for (**a**) a highly reduced composition of gases, including CH${}_{4}$, NH${}_{3}$ and H${}_{2}$O in a ratio of 0.7:0.2:0.1 and (**b**) a mildly reduced composition of gases, including N${}_{2}$, CO${}_{2}$, CO, H${}_{2}$O and H${}_{2}$ in the ratio of 0.7:0.2 for N${}_{2}$/CO${}_{2}$ with traces of CO, H${}_{2}$O and H${}_{2}$ in fractions of 0.05, 0.025 and 0.025, respectively.

MCF simulations have been performed for different values of the reduced electric field, namely 0.1, 10, 20 and 50 Td (1 Td = 10${}^{-21}$ V m${}^{2}$). The population of rotational and vibrational levels is calculated according to a Boltzmann distribution at 300 K. Zeroth ($f_{0}$) and first order ($f_{1}$) Legendre polynomials coefficients calculated from MCF are shown in Figure 6 for the highly reduced and mildly reduced mixture of gases.

In both conditions, the simulated EEDFs show strong deviations from a Boltzmann equilibrium distribution, due to the several electron–molecules interactions that are taken into account. In particular, in Figure 6a, an energetic tail of the EEDF is obtained for energies above 1 eV. This is due to the presence of a deep Ramsauer-Townsend minimum in the elastic momentum transfer cross section of CH${}_{4}$, that is the dominant gas of this mixture. For energies lower than 1 eV, electron impact rotational collisions with water molecules are dominant and lead to a peak in the body of the EEDFs. In Figure 6b, a more structured EEDF is obtained with the presence of plateaux and drops in the distributions. This feature is typical of molecular gases, such as N${}_{2}$ and CO${}_{2}$, that present resonant vibrational processes. In fact, those inelastic processes act as a sharp barrier that electrons have to overcome to reach higher energies [91]. Moreover, in this case, the magnitude of the first Legendre polynomial coefficient of the EVDF expansion ($f_{1}$) is comparable with the zeroth order one ($f_{0}$). This indicates that the popular two-term approximation that is employed in many codes (e.g., in [64,80]) would not be accurate for calculations of steady-state EVDFs.

In both cases, the calculated distribution of kinetic energy differs by orders of magnitude, in specific regions, from the Maxwell Boltzmann distribution. We therefore already have the certainty, in extreme cases of composition, that the calculation of the specific reaction rates performed with traditional methods of the primary processes of Miller experience is totally inadequate.

To summarize, calculations show that: (*i*) a strong deviation from the equilibrium distributions is obtained considering both a Miller-Urey-type composition of gases and a more realistic primordial Earth atmosphere; (ii) computationally efficient calculations can be performed using MCF, where great details are included in the molecular physics of vibrational and rotational states; (iii) consistent and complete sets of cross sections can be gathered from the literature to study the electron kinetics in gas mixtures relevant for the interpretation of Miller-Urey’s experiment as well as its modern variants.

Future work should be focused on coupling the MCF method with other codes that calculate the non-equilibrium vibrational distribution function of the molecules, such as the Fokker-Planck method that has been introduced before.

## 4. Quantum Calculations for Elementary Processes

As emerged in Section 2 and Section 3, a crucial point that may facilitate the communication between the two disciplines — prebiotic chemistry and plasma kinetics — relies on the abundance of molecular data and the knowledge of reaction rates. As we have pointed out, kinetic studies related to Miller’s experience have so far largely underestimated the fact that the primary events in this kind of experiments are induced by impacts of molecules with electrons and other high energy secondary particles produced ultimately by the presence of a strong electric field. On the other hand, these aspects have always played a central role in the study of plasma reactors.

In the plasma simulation community, the problem of availability of cross sections and specific reaction rates of elementary processes emerged and has been constantly considered for decades. This has emerged also very clearly from the discussion of the cross sections used for EVDF calculations in the previous section. A constant dialogue with the elementary process community is producing new sets of data optimized for the use with specific plasma modeling methods.

As a consequence of this virtuous long-term collaboration, in the last few years extremely effective and rigorous calculation methods have been developed—based on quantum mechanical foundations of the processes—that allow one to calculate cross sections of excitation and ionization processes for chemical species of any reasonable complexity in the context of plasma sources. Currently, numerous databases relating to these processes have been developed—see for example the KIDA database for astrochemistry reactions [93], the HITRAN database for molecular spectroscopy [94], the LXCat database for plasma modeling [95] and ExoMol database for exoplanet atmospheres [96]—and constantly updated. They are managed by many active research groups in mutual communication.

It is not possible to describe in details here the different methods that have proven to be the most effective. In general, they range from relatively elementary and ready-to-use techniques, based for example on improvements of the Born approximation, to very advanced techniques that solve fine details of quantum aspects of the interaction between the particles involved. In particular, these advanced methods are promising when applied to reviewing historical experiences on the origin of chemical species of biological interest; as to physical rigor, they are characterized by an appropriate versatility, that is useful not only for the simpler aspects related to primary events occurring in plasma (excitation and ionization of small molecules), but also for the analysis of relatively simpler chemical processes among those which involve species of biological interest.

Among the advanced techniques, the R-matrix method, developed in the field of astrochemistry by the Tennyson’s group [97], with which one of the authors of this work has recently collaborated, is particularly interesting. In the R-matrix approach, the space around the molecule is divided into an inner and an outer region. The methodology is based on the self-consistency of two solutions of the wave equations with many particles in these two regions: (*i*) in the inner part, related to interactions occurring at short distances, full ab-initio quantum chemistry calculations are used, whereas (ii) in the outer region, only effective long range interactions are taken into account.

However, methods like the R-matrix can give information only for a given frozen configuration of the molecule, normally at equilibrium geometry. But this is not enough in cases where vibrations and rotations of molecules must be taken into account. This is the case of non-equilibrium systems. In order to take in account internal degrees of freedom, other techniques have to be considered, such as the Local-complex-potential (LCP) [98] and the Multichannel-Quantum-Defect-theory (MQDT) [99].

**Figure 7:**
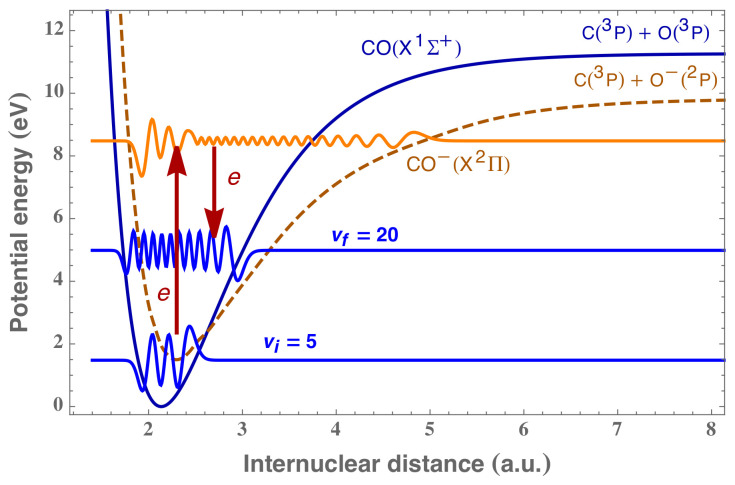
Schematic representation of the vibrational excitation process by electron impact in the case of a CO molecule: in blue, the initial ($v_{i}=5$) and final ($v_{f}=20$) vibrational wave functions of CO are represented. The resonant CO${}^{-}$ wave function is reported in orange.

As an example of a relatively simple application, the LCP method can be used for the vibrational excitation of the diatomic CO-type (relevant for the kinetics of Miller-type experiences); there are no special difficulties in applying the same technique to different chemical processes and to more complex molecules such as water, ammonia, and even simple amino acids. Figure 7 reports the potential energy curves for CO and the corresponding resonant state CO${}^{-}$ as a function of the internuclear distance *R* and schematically represents the vibrational excitation reaction:

(9) $$e+\mathrm{CO}\left(X,v_{i}=5\right)\to {\mathrm{CO}}^{-}\to e+\mathrm{CO}\left(X,v_{f}=20\right)\;,$$

where the CO molecule is supposed to be in the initial vibrational state $v_{i}=5$ and the incoming electron to have an energy of 7 eV. The system then proceeds with a sequence of two steps: (i) the electron is captured into the target, the system then jumps from the initial wave function towards the resonant state; (ii) the resonant state is metastable for some time and decays into the final vibrationally excited CO and ejects an electron. If the potential energy curves and the couplings between the target CO molecule and the resonant CO${}^{-}$ state were known, it would be possible to determine the cross sections of the process.

**Figure 8:**
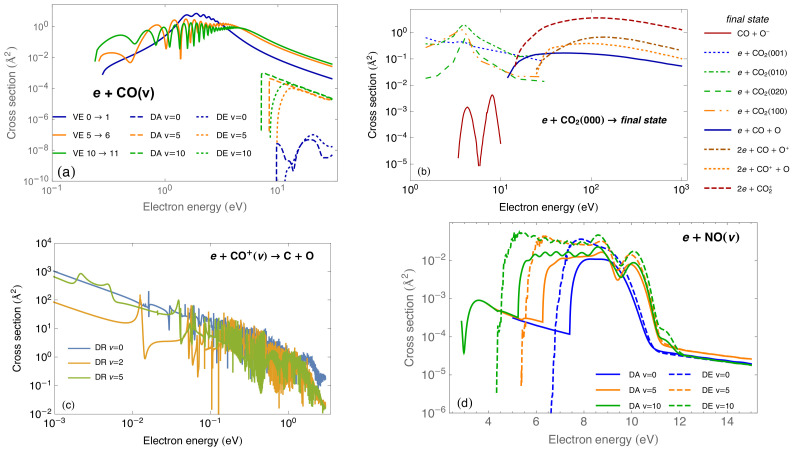
Compilation of state-resolved cross sections by electron-impact: (**a**) electron-CO [100,101]; (**b**) electron-CO${}_{2}$ [105]; (**c**) electron-CO${}^{+}$ [104]; (**d**) electron-NO [102,103]. Processes of: Vibrational Excitation (VE); Dissociative Attachment (DA); Dissociative Recombination (DR); Dissociative Excitation (DE).

In recent years, motivated by the great importance of CO, CO${}_{2}$, CO${}^{+}$ and NO molecules in astrophysics, aerospace and laser technologies, great improvements, theoretically and experimentally, in the quantitative understanding of the reactivity of these species in a weakly-ionized plasma have been achieved. Figure 8 summarizes the results for cross sections by electron impact for CO [100,101] and NO [102,103] molecules obtained with the LCP method, for CO${}^{+}$ [104] molecular ion obtained in the MQDT framework and for experimental data for the CO${}_{2}$ molecule [105].

An important aspect of these studies, and generally of all modeling studies on plasma kinetics, is the validation. This is a complex question because many details of the cross sections and kinetics revealed by these models are difficult to verify by direct experimental investigation. As far as cross sections are concerned, the most common procedure is verifying the set of cross sections as a whole, by comparing the transport quantities calculated by means of the type of calculations described in previous section. Since these calculations depend on parameters, such as the reduced electric field, which are easily modifiable in a wide range both in the model and in the experiment, the possibility of validation is concrete [106,107]. Several techniques in development, including the possibility of employing Machine Learning methods, promise to make progress in this type of procedure [108].

## 5. Conclusions and Perspectives

In the present paper, it has been shown that the methods used in the plasma modeling community reveal crucial aspects of the chemical processes that have never been taken into account in previous studies of prebiotic processes. Actually, the energy and population distribution deviate considerably from the prediction of traditional statistical mechanics, and the reaction rates are strongly dependent on such distributions. Thereby, we propose to apply the theoretical methodologies that have been developed for the study of ionized gases to the Miller-Urey experience. In this regard, in the last decades, many so-called self-consistent models were developed in the field of plasma kinetics, which can reliably calculate the kinetics of the species produced in the plasma starting from physical conditions in the reactor. Therefore, in the light of real progress in the field of prebiotic chemistry, the first step consists of determining the conditions of the spark region from the point of view of the plasma physics and chemistry: the ionization degree of the gas, the energy distribution of free electrons and the formation of the simplest reactive species that underlie the complex chemical network.

In conclusion, in this paper, we have presented a new perspective on future research on the chemical kinetics of the first active species in the primordial atmosphere and their subsequent role in the formation of the first prebiotic species. The framework of this study is the Miller-Urey experiment, its improvement and understanding, and the clues it provides into the possibility that life machinery arose as a result of an abiotic process. Two new elements of knowledge may produce a synergistic push towards further progress: the first is the awareness that the primordial atmosphere was probably not the strongly reducing mixture with essentially solar-nebula composition it was believed to be in Miller’s time. The second is the development of new methods in the context of the computer modeling of the kinetics of plasmas, motivated by ecological, astrophysical and aerospace problems. The communication between the two communities of plasma kinetics and prebiotic chemistry can therefore help, in the future, to attain a better understanding and new insight into the chemical kinetics of a historical experiment, which has changed our ideas on the genesis of prebiotic molecules on the primordial Earth.

## References

[1] Chang S., DesMarais D., Mack R., Miller S., Strathearn G. (1983). Prebiotic Organic Syntheses and the Origin of Life, in Earth’s Earliest Atmospheres.

[2] Lazcano A., Miller S.L. (1996). The origin and early evolution of life: Prebiotic chemistry, the pre-RNA world, and time. Cell.

[3] Miller S.L., Cleaves H.J. (2006). Prebiotic chemistry on the primitive Earth. Syst. Biol..

[4] Ehrenfreund P., Rasmussen S., Cleaves J., Chen L. (2006). Experimentally tracing the key steps in the origin of life: The aromatic world. Astrobiology.

[5] Chyba C., Sagan C. (1992). Endogenous production, exogenous delivery and impact-shock synthesis of organic molecules: An inventory for the origins of life. Nature.

[6] Bernstein M. (2006). Prebiotic materials from on and off the early Earth. Philos. Trans. R. Soc. B Biol. Sci..

[7] Maurette M., Brack A., Kurat G., Perreau M., Engrand C. (1995). Were micrometeorites a source of prebiotic molecules on the early Earth?. Adv. Space Res..

[8] Ehrenfreund P., Irvine W., Becker L., Blank J., Brucato J., Colangeli L., Derenne S., Despois D., Dutrey A., Fraaije H. (2002). Astrophysical and astrochemical insights into the origin of life. Rep. Prog. Phys..

[9] Burchell M.J. (2004). Panspermia today. Int. J. Astrobiol..

[10] Coulson S. (2004). On panspermia and the survivability of micrometre-sized meteoroids within the Earth’s atmosphere. Int. J. Astrobiol..

[11] Flynn G., Keller L., Jacobsen C., Wirick S. (2004). An assessment of the amount and types of organic matter contributed to the Earth by interplanetary dust. Adv. Space Res..

[12] Micca Longo G., Longo S. (2018). Theoretical analysis of the atmospheric entry of sub-mm meteoroids of Mg_x_Ca_1-x_CO_3_ composition. Icarus.

[13] Micca Longo G., Piccinni V., Longo S. (2019). Evaluation of CaSO_4_ micrograins in the context of organic matter delivery: Thermochemistry and atmospheric entry. Int. J. Astrobiol..

[14] Micca Longo G., D’Elia M., Fonti S., Longo S., Mancarella F., Orofino V. (2019). Kinetics of White Soft Minerals (WSMs) Decomposition under Conditions of Interest for Astrobiology: A Theoretical and Experimental Study. Geosciences.

[15] Matrajt G., Messenger S., Brownlee D., Joswiak D. (2012). Diverse forms of primordial organic matter identified in interplanetary dust particles. Meteorit. Planet. Sci..

[16] Yabuta H., Uesugi M., Naraoka H., Ito M., Kilcoyne A.L.D., Sandford S.A., Kitajima F., Mita H., Takano Y., Yada T. (2014). X-ray absorption near edge structure spectroscopic study of Hayabusa category 3 carbonaceous particles. Earth Planets Space.

[17] Dong H., Rech J.A., Jiang H., Sun H., Buck B.J. (2007). Endolithic cyanobacteria in soil gypsum: Occurrences in Atacama (Chile), Mojave (United States), and Al-Jafr Basin (Jordan) Deserts. J. Geophys. Res. Biogeosci..

[18] Gooding J.L., Wentworth S.J., Zolensky M.E. (1988). Calcium carbonate and sulfate of possible extraterrestrial origin in the EETA 79001 meteorite. Geochim. Cosmochim. Acta.

[19] Micca Longo G., Longo S. (2020). The role of primordial atmosphere composition in organic matter delivery to early Earth. Rend. Lincei. Sci. Fis. E Nat..

[20] Miller S.L. (1953). A Production of Amino Acids Under Possible Primitive Earth Conditions. Science.

[21] Miller S.L. (1955). Production of Some Organic Compounds under Possible Primitive Earth Conditions1. J. Am. Chem. Soc..

[22] Miller S.L., Urey H.C. (1959). Organic Compound Synthes on the Primitive Eart. Science.

[23] Oparin A. (1924). Proischogdenie Zhizni.

[24] Marshall W.L. (1994). Hydrothermal synthesis of amino acids. Geochim. Cosmochim. Acta.

[25] Lazcano A., Bada J.L. (2003). The 1953 Stanley L. Miller Experiment: Fifty Years of Prebiotic Organic Chemistry. Orig. Life Evol. Biosph..

[26] Trainer M.G., Pavlov A.A., Curtis D.B., Mckay C.P., Worsnop D.R., Delia A.E., Toohey D.W., Toon O.B., Tolbert M.A. (2004). Haze aerosols in the atmosphere of early Earth: Manna from heaven. Astrobiology.

[27] Hasenkopf C.A., Freedman M.A., Beaver M.R., Toon O.B., Tolbert M.A. (2011). Potential climatic impact of organic haze on early Earth. Astrobiology.

[28] Balucani N. (2009). Elementary reactions and their role in gas-phase prebiotic chemistry. Int. J. Mol. Sci..

[29] Bada J.L. (2013). New insights into prebiotic chemistry from Stanley Miller’s spark discharge experiments. Chem. Soc. Rev..

[30] Haldane J.B.S. (1933). Sciende and Human Life.

[31] Hart M.H. (1978). The evolution of the atmosphere of the earth. Icarus.

[32] Holland H.D. (1962). Petrologic Studies: A Volume to Honor A.F. Buddington.

[33] Abelson P.H. (1966). Chemical Events on the Primitive Earth. Proc. Natl. Acad. Sci. USA.

[34] Holland H. (1984). The Chemical Evolution of the Atmosphere and Oceans.

[35] Kasting J.F. (1993). Earth’s early atmosphere. Science.

[36] Schlesinger G., Miller S.L. (1983). Prebiotic synthesis in atmospheres containing CH4, CO, and CO2. J. Mol. Evol..

[37] Miyakawa S., Yamanashi H., Kobayashi K., Cleaves H.J., Miller S.L. (2002). Prebiotic synthesis from CO atmospheres: Implications for the origins of life. Proc. Natl. Acad. Sci. USA.

[38] Cleaves H.J., Chalmers J.H., Lazcano A., Miller S.L., Bada J.L. (2008). A Reassessment of Prebiotic Organic Synthesis in Neutral Planetary Atmospheres. Orig. Life Evol. Biosph..

[39] Zahnle K., Schaefer L., Fegley B. (2010). Earth’s Earliest Atmospheres. Cold Spring Harb. Perspect. Biol..

[40] Saitta A.M., Saija F. (2014). Miller experiments in atomistic computer simulations. Proc. Natl. Acad. Sci. USA.

[41] Pietrucci F., Saitta A.M. (2015). Formamide reaction network in gas phase and solution via a unified theoretical approach: Toward a reconciliation of different prebiotic scenarios. Proc. Natl. Acad. Sci. USA.

[42] Ferus M., Pietrucci F., Saitta A.M., Knížek A., Kubelík P., Ivanek O., Shestivska V., Civiš S. (2017). Formation of nucleobases in a Miller–Urey reducing atmosphere. Proc. Natl. Acad. Sci. USA.

[43] Langmuir I. (1928). Oscillations in ionized gases. Proc. Natl. Acad. Sci. USA.

[44] Reid Thompson W., Henry T.J., Schwartz J.M., Khare B., Sagan C. (1991). Plasma discharge in N_2_ + CH_4_ at low pressures: Experimental results and applications to Titan. Icarus.

[45] Alcouffe G., Cavarroc M., Cernogora G., Ouni F., Jolly A., Boufendi L., Szopa C. (2009). Capacitively coupled plasma used to simulate Titan’s atmospheric chemistry. Plasma Sources Sci. Technol..

[46] Sciamma-O’Brien E., Ricketts C.L., Salama F. (2014). The Titan Haze Simulation experiment on COSmIC: Probing Titan’s atmospheric chemistry at low temperature. Icarus.

[47] Dubois D., Carrasco N., Jovanovic L., Vettier L., Gautier T., Westlake J. (2020). Positive ion chemistry in an N_2_-CH_4_ plasma discharge: Key precursors to the growth of Titan tholins. Icarus.

[48] Köhn C., Dujko S., Chanrion O., Neubert T. (2019). Streamer propagation in the atmosphere of Titan and other N_2_:CH_4_ mixtures compared to N_2_:O_2_ mixtures. Icarus.

[49] Dubrovin D., Nijdam S., van Veldhuizen E.M., Ebert U., Yair Y., Price C. (2010). Sprite discharges on Venus and Jupiter-like planets: A laboratory investigation. J. Geophys. Res. Space Phys..

[50] Libby W.F. (1979). Plasma chemistry. J. Vac. Sci. Technol..

[51] Gicquel A., Silva F., Hassouni K. (2000). Diamond Growth Mechanisms in Various Environments. J. Electrochem. Soc..

[52] Herrebout D., Bogaerts A., Yan M., Gijbels R., Goedheer W., Dekempeneer E. (2001). One-dimensional fluid model for an rf methane plasma of interest in deposition of diamond-like carbon layers. J. Appl. Phys..

[53] Okita A., Suda Y., Oda A., Nakamura J., Ozeki A., Bhattacharyya K., Sugawara H., Sakai Y. (2007). Effects of hydrogen on carbon nanotube formation in CH4/H2 plasmas. Carbon.

[54] (2011). Physical Kinetics of Ionized Gases. Fundamentals of Ionized Gases.

[55] Meek J.M., Craggs J.D. (1953). Electrical Breakdown of Gases.

[56] Von Engel A. (1983). Electric Plasmas-Their Nature and Uses.

[57] Williams E.R., Geotis S.G., Bhattacharya A. (1989). A radar study of the plasma and geometry of lightning. J. Atmos. Sci..

[58] Braglia G. (1977). The diffusion and drift of electrons in gases a Monte-Carlo simulation. Phys. B+ C.

[59] Schaefer G., Hui P. (1990). The Monte Carlo flux method. J. Comput. Phys..

[60] Bogaerts A., Gijbels R. (1996). Mathematical description of a direct current glow discharge in argon. Fresenius’ J. Anal. Chem..

[61] Biagi S.F. (1999). Monte Carlo simulation of electron drift and diffusion in counting gases under the influence of electric and magnetic fields. Nucl. Instrum. Methods Phys. Res. Sect. A Accel. Spectrometers Detect. Assoc. Equip..

[62] Longo S. (2000). Monte Carlo models of electron and ion transport in non-equilibrium plasmas. Plasma Sources Sci. Technol..

[63] Loffhagen D., Winkler R., Donkó Z. (2002). Boltzmann equation and Monte Carlo analysis of the spatiotemporal electron relaxation in nonisothermal plasmas. Eur. Phys. J. Appl. Phys..

[64] Hagelaar G.J.M., Pitchford L.C. (2005). Solving the Boltzmann equation to obtain electron transport coefficients and rate coefficients for fluid models. Plasma Sources Sci. Technol..

[65] Capitelli M., Celiberto R., Colonna G., Esposito F., Gorse C., Hassouni K., Laricchiuta A., Longo S. (2015). Fundamental Aspects of Plasma Chemical Physics: Kinetics.

[66] Vialetto L., Longo S., Diomede P. (2019). Benchmark calculations for electron velocity distribution function obtained with Monte Carlo Flux simulations. Plasma Sources Sci. Technol..

[67] Gordiets B.F., Osipov A.I., Stupochenko E.V., Shelepin L.A. (1973). Vibrational Relaxation in Gases and Molecular Lasers. Sov. Phys. Uspekhi.

[68] Capitelli M., Armenise I., Bruno D., Cacciatore M., Celiberto R., Colonna G., Pascale O.D., Diomede P., Esposito F., Gorse C. (2007). Non-equilibrium plasma kinetics: A state-to-state approach. Plasma Sources Sci. Technol..

[69] Laux C.O., Pierrot L., Gessman R.J. (2012). State-to-state modeling of a recombining nitrogen plasma experiment. Chem. Phys..

[70] Kim J.G., Boyd I.D. (2013). State-resolved master equation analysis of thermochemical nonequilibrium of nitrogen. Chem. Phys..

[71] Armenise I., Kustova E. (2013). State-to-state models for CO2 molecules: From the theory to an application to hypersonic boundary layers. Chem. Phys..

[72] Kadochnikov I.N., Arsentiev I.V. (2018). Kinetics of nonequilibrium processes in air plasma formed behind shock waves: State-to-state consideration. J. Phys. D: Appl. Phys..

[73] Guerra V., Silva T., Ogloblina P., Grofulović M., Terraz L., da Silva M.L., Pintassilgo C.D., Alves L.L., Guaitella O. (2017). The case for in situ resource utilisation for oxygen production on Mars by non-equilibrium plasmas. Plasma Sources Sci. Technol..

[74] Pietanza L.D., Colonna G., D’Ammando G., Laricchiuta A., Capitelli M. (2015). Vibrational excitation and dissociation mechanisms of CO2 under non-equilibrium discharge and post-discharge conditions. Plasma Sources Sci. Technol..

[75] Rusanov V.D., Fridman A.A., Sholin G.V. (1981). The physics of a chemically active plasma with nonequilibrium vibrational excitation of molecules. Sov. Phys. Uspekhi.

[76] Viegas P., van de Sanden M.C.M., Longo S., Diomede P. (2019). Validation of the Fokker–Planck Approach to Vibrational Kinetics in CO2 Plasma. J. Phys. Chem. C.

[77] Longo S., van de Sanden M.C.M., Diomede P. (2019). Fokker–Planck equation for chemical reactions in plasmas. Rend. Lincei. Sci. Fis. E Nat..

[78] Diomede P., van de Sanden M.C.M., Longo S. (2018). Vibrational Kinetics in Plasma as a Functional Problem: A Flux-Matching Approach. J. Phys. Chem. A.

[79] Petrović Z.L., Dujko S., Marić D., Malović G., Nikitović Ž., Šašić O., Jovanović J., Stojanović V., Radmilović-Radjenović M. (2009). Measurement and interpretation of swarm parameters and their application in plasma modelling. J. Phys. D Appl. Phys..

[80] Tejero-del-Caz A., Guerra V., Gonçalves D., da Silva M.L., Marques L., Pinhão N., Pintassilgo C.D., Alves L.L. (2019). The LisbOn KInetics Boltzmann solver. Plasma Sources Sci. Technol..

[81] Stephens J. (2018). A multi-term Boltzmann equation benchmark of electron-argon cross-sections for use in low temperature plasma models. J. Phys. D Appl. Phys..

[82] Rabie M., Franck C.M. (2016). METHES: A Monte Carlo collision code for the simulation of electron transport in low temperature plasmas. Comput. Phys. Comm..

[83] Vialetto L., Viegas P., Longo S., Diomede P. (2020). Benchmarking of Monte Carlo Flux simulations of electrons in CO_2_. Plasma Sources Sci. Technol..

[84] McCollom T.M. (2013). Miller-Urey and beyond: What have we learned about prebiotic organic synthesis reactions in the past 60 years?. Annu. Rev. Earth Planet. Sci..

[85] Scherer S., Wollrab E., Codutti L., Carlomagno T., da Costa S.G., Volkmer A., Bronja A., Schmitz O.J., Ott A. (2017). Chemical analysis of a “Miller-type” complex prebiotic broth. Orig. Life Evol. Biosph..

[86] Fowler R.H. (1924). XXIII. Statistical equilibrium with special reference to the mechanism of ionization by electronic impacts. Lond. Edinb. Dublin Philos. Mag. J. Sci..

[87] Magboltz - Transport of Electrons in Gas Mixtures. http://magboltz.web.cern.ch/magboltz/.

[88] Hayashi Database. www.lxcat.net.

[89] Biagi Database. www.lxcat.net.

[90] Vialetto L., Guerra V., Diomede P., Longo S., Ben Moussa A., van Dijk J., Alves L.L. (2021). Effect of anisotropic scattering on electron transport parameters in CO. Plasma Sources Sci. Technol..

[91] Capitelli M., Ferreira C.M., Gordiets B.F., Osipov A.I. (2013). Plasma Kinetics in Atmospheric Gases.

[92] IST-Lisbon Database. www.lxcat.net.

[93] Wakelam V., Loison J.C., Herbst E., Pavone B., Bergeat A., Béroff K., Chabot M., Faure A., Galli D., Geppert W.D. (2015). The 2014 KIDA Network for Interstellar Chemistry. Astrophys. J. Suppl. Ser..

[94] Gordon I., Rothman L., Hill C., Kochanov R., Tan Y., Bernath P., Birk M., Boudon V., Campargue A., Chance K. (2017). The HITRAN2016 molecular spectroscopic database. J. Quant. Spectrosc. Radiat. Transf..

[95] Pitchford L.C., Alves L.L., Bartschat K., Biagi S.F., Bordage M.C., Bray I., Brion C.E., Brunger M.J., Campbell L., Chachereau A. (2016). LXCat: An Open-Access, Web-Based Platform for Data Needed for Modeling Low Temperature Plasmas. Plasma Process. Polym..

[96] Tennyson J., Yurchenko S.N., Al-Refaie A.F., Barton E.J., Chubb K.L., Coles P.A., Diamantopoulou S., Gorman M.N., Hill C., Lam A.Z. (2016). The ExoMol database: Molecular line lists for exoplanet and other hot atmospheres. J. Mol. Spectrosc..

[97] Tennyson J. (2010). Electron-molecule collision calculations using the *R*-matrix method. Phys. Rep..

[98] Giusti-Suzor A., Bardsley J.N., Derkits C. (1983). Dissociative recombination in low-energy *e*-H2+ collisions. Phys. Rev. A.

[99] Seaton M.J. (1983). Quantum defect theory. Rep. Prog. Phys..

[100] Laporta V., Cassidy C.M., Tennyson J., Celiberto R. (2012). Electron-impact resonant vibration excitation cross sections and rate coefficients for carbon monoxide. Plasma Sources Sci. Technol..

[101] Laporta V., Tennyson J., Celiberto R. (2016). Carbon monoxide dissociative attachment and resonant dissociation by electron-impact. Plasma Sources Sci. Technol..

[102] Laporta V., Tennyson J., Schneider I.F. (2020). Vibrationally resolved NO dissociative excitation cross sections by electron impact. Plasma Sources Sci. Technol..

[103] Laporta V., Schneider I.F., Tennyson J. (2020). Dissociative electron attachment cross sections for ro-vibrationally excited NO molecule and N- anion formation. Plasma Sources Sci. Technol..

[104] Moulane Y., Mezei J.Z., Laporta V., Jehin E., Benkhaldoun Z., Schneider I.F. (2018). Reactive collision of electrons with CO+ in cometary coma. Astron. Astrophys..

[105] Itikawa Y. (2002). Cross Sections for Electron Collisions With Carbon Dioxide. J. Phys. Chem. Ref. Data.

[106] Rockwood S.D. (1973). Elastic and inelastic cross sections for electron-Hg scattering from Hg transport data. Phys. Rev. A.

[107] Phelps A.V., Pitchford L.C. (1985). Anisotropic scattering of electrons by N_2_ and its effect on electron transport. Phys. Rev. A.

[108] Morgan W.L. (1991). The feasibility of using neural networks to obtain cross sections from electron swarm data. IEEE Trans. Plasma Sci..

